# The effect of the housing crisis in the Alabama Black Belt on respiratory health

**DOI:** 10.3389/falgy.2024.1413171

**Published:** 2024-08-21

**Authors:** Sharlene D. Newman, Aylin Akca Sumengen, Michael Rasbury, Steven McDaniel

**Affiliations:** ^1^ Alabama Life Research Institute, The University of Alabama, Tuscaloosa, AL, United States; ^2^ Capstone College of Nursing, The University of Alabama, Tuscaloosa, AL, United States; ^3^ Alabama Safe State, College of Engineering, The University of Alabama, Tuscaloosa, AL, United States; ^4^ Alabama Department of Public Health, Montgomery, AL, United States

**Keywords:** housing, asthma, manufactured homes, rural, allergies

## Abstract

**Background:**

There is a growing housing crisis in rural America with homelessness growing in addition to a growing number of substandard homes due to an inability to afford the costs of repair and maintenance. The goal of the current study was to assess the housing concerns in rural Alabama Black Belt communities which are often understudied and the relationship between housing quality and respiratory health.

**Methods:**

A semi-random sampling of five Black Belt counties was conducted to obtain a sample of 253 rural households. The survey was designed to obtain information regarding household income, housing status including a list of safety concerns and respiratory health. A *χ*^2^ analysis was performed to examine the effect of housing type and income on prevalence of respiratory illness and safety home concerns (e.g., roofing, windows/doors, floors, mold/mildew).

**Results:**

The majority of households surveyed had an annual income below $15,000 and owned their homes with over half of the homes being manufactured homes. Lower income was associated with increased prevalence of asthma [*χ*^2^(2, *N* = 237) = 7.75, *p* = 0.021], while living in a manufactured home was associated with increased risk of allergies [*χ*^2^(1, *N* = 251) = 7.88, *p* = 0.005]. Additionally, poor windows and doors [*χ*^2^(1, *N* = 253) = 3.8, *p* = 0.05] was associated with higher prevalence of asthma.

**Conclusions:**

The results confirm and expand previous results and demonstrate the relationship between quality housing and allergy and asthma prevalence in rural areas with an abundance of aging manufactured homes.

## Introduction

The Alabama Black Belt region was significantly impacted by the COVID-19 pandemic with it having a 30% higher death rate than non-Black Belt counties in the state in 2020 (1). While many studies have focused on the lack of access to healthcare (2), the housing conditions in the region may have also played a role in the spread and deathly effect of the respiratory virus. In recent years it has become clear that housing is healthcare. This is not just true with respect to homelessness (3) but also for individuals living in substandard housing (4).

Housing conditions have long been linked to respiratory illnesses including asthma and allergies (5) with poor housing quality being associated with risk of childhood asthma and asthma morbidity (6, 7). A potential reason for this link is that poor housing quality is associated with increased risk of mold (8) which is linked to the prevalence of childhood wheezing and asthma (9, 10). In addition to housing quality, income has also consistently been shown to be associated with asthma prevalence and severity (11). For example, the Centers for Disease Control and Prevention reported an association between asthma prevalence and poverty level with the prevalence being 10.4% for household income below 100% of the poverty level but only 6.8% for household income 450% of the poverty level and above (12). It should be noted that income influences housing quality, making the Black Belt region susceptible to high rates of housing quality issues which likely contributes to health disparities.

Based on 2021 CDC data, the prevalence rate of asthma in adults in the U.S. is 8%. Alabama has a moderately high prevalence rate of adult asthma (10.1% for adults, ranked 21st highest out of 50 states) but has a high asthma mortality rate (ranked 7th highest out of 50 states) (8). We hypothesized that poor quality housing and income are associated with respiratory health issues (asthma and allergies) in Alabama Black Belt counties. We focus on the Black Belt region for several reasons. First, the soil in the region is nonporous. After the Civil War when Black citizens could purchase land, they were often sold the worse land (13, 14). Cotton was farmed at higher elevations with better drainage while less productive lowland areas were the areas where black residents were able to acquire land (7). These lowland areas are more likely to flood and have standing water for days after a rain. The water that accumulates on the surface after a rain can cause damage to the foundation of homes, increase mold growth, increase pests including mosquitoes among other things.

In addition to the problems caused by the soil, the 1968 Fair Housing Act disproportionately resulted in residents in the Black Belt having substandard housing today. Section 235 of the Fair Housing Act supported and encouraged new home construction. However, the federal government failed to manage the program. As a result, many of the newly constructed homes, particularly in Black neighborhoods, had poor workmanship and were in immediate need of repair (15). For example, in one Black Belt town, Camden, it was reported that the houses built via the Fair Housing Act in white neighborhoods were better built brick houses while those built in Black neighborhoods used substandard materials and had construction problems from the outset (16). Many of the homes were abandoned and replaced with less expensive manufactured homes (7).

Manufactured housing, particularly mobile homes, is used extensively in the Deep South. A previous report found that over 20% of the residents in the Alabama Black Belt live in mobile homes with 17% of them being built before 1979 (17). Mobile homes are popular for several reasons. First, they are more affordable which allows for homeownership. This is important because there are few rentals in rural areas; the owner-occupied rate in the region per county ranges from 70–80+% (U.S. Census). Also, many individuals have family or heirs property. Heirs property is difficult to build homes on because it is not possible to get a mortgage due to an inability to obtain a deed (18). It is unclear how these manufactured homes age and potentially affect respiratory health, particularly in lowland areas with standing water problems.

Finally, due to the history of the region including the racial injustice perpetrated there, over 52% of the residents are African American and there is persistent poverty with the counties that make up the Black Belt having the highest poverty rates in the state and country (19). This persistent poverty creates a population without the means to repair and mitigate the damage caused by the nonporous soil that is potentially creating damage to the homes or properly repair ill constructed homes via the Fair Housing Act.

There are limited studies of the relationship between housing quality and asthma and allergy prevalence in the Black Belt region. In this initial small-scale study we attempt to obtain a better understanding of the influence of housing conditions on the high prevalence of asthma rates in the region. There are also limited studies examining manufactured homes. Therefore, this study fills an important gap.

## Methods

### Survey methodology

#### Population characteristics and sample design

The study was designed to establish an association between housing conditions and respiratory disease prevalence among rural Alabama Black Belt communities. Semi-random sampling was conducted to obtain a sample of 253 rural households from five Alabama Black Belt counties—Choctaw, Lowndes, Marengo, Sumter, and Wilcox. The communities selected were part of the target area for a Housing and Urban Development (HUD) grant (see Table 1 for county information). The study was approved by The University of Alabama Institutional Review Board and met all ethical standards.

**Table 1:** County information.

County	Pre-1978 Housing units	Pre-1978 Owner occupied (#)	Median household income	Poverty rate %	# Housing units
Choctaw	2,354	1,821	$35,892	22.6	12,792
Lowndes	1,308	968	$30,036	26.6	4,833
Marengo	3,484	2,374	$33,241	24.8	9,850
Sumter	2,117	1,348	$24,320	36.4	6,340
Wilcox	1,314	985	$31,014	32.5	5,304

#### Data collection and analysis

Surveys were distributed via a website and at community events. Surveys were returned via email and transcribed into a spreadsheet. The survey obtained the following information: home address, whether the family owned or rented the home, whether they lived in a manufactured/mobile home, total annual household income, number of people in the household, number of children in the household, whether there were individuals over age 65 or disabled in the household, the health concerns of individuals in the household, and the safety concerns of the home.

Scores for prevalence of asthma and allergies among household members, home ownership and housing type (i.e., manufactured homes) were analyzed using descriptive frequency scores. Assessment of housing conditions was based on participants' listing of problems with the home. The frequency of the mention of the following terms were calculated: mold or mildew; roof; and sewer, sewage or wastewater. Finally, a chi-square analysis was performed to examine differences in measures by housing type and income.

## Results

### Summary of data

As shown in Table 2, the majority of households surveyed had a total annual income below $15,000, included an individual 65 years old or older and with 42.2% of the households including an individual with a disability. 36% of the households had at least one child (5% did not respond).

**Table 2:** Descriptive data.

Rent Home	5.60%
Mobile Homes	51.40%
# children in home	0.68 ± 1.08
Mold/Mildew	21.70%
Roof problems	36.40%
Windows/Doors drafty	40%
Floors/foundation problems	38.7%
Sewage problems	10.30%
65+	61.10%
Disabled	42.20%
Asthma	31.60%
Allergies	56.90%
Depression or anxiety	26.10%
Annual Income	
Less than $15,000	53.7%
$15,000–$30,000	32.0%
$30,000–$50,000	7.9%
Greater than $50,000	1.2%
Did not answer	5.1%

The majority of residents owned their homes with only 5.6% renting. Over half of the homes were manufactured homes. At least one structural issue was reported in approximately 40% of homes (e.g., windows/doors, roof, floor/foundations). Mold and/or mildew was reported in 21.7% of homes and sewage/wastewater issues in 10.3%.

With respect to health, the majority of the households reported at least one member having problems with allergies, with 31.6% having at least one household member with asthma.

### Statistical analysis

A *χ*^2^ analysis was performed to examine the frequency of having/not having asthma and allergies and housing problems as a function of dwelling type (mobile home) and income. Living in a mobile home increased the likelihood of allergies [*χ*^2^(1, *N* = 251) = 7.88, *p* = 0.005], asthma [*χ*^2^(1,*N* = 251) = 3.56, *p* = 0.059], and problems with floors/foundations [*χ*^2^(1,*N* = 251) = 6.3, *p* = 0.012] (see Supplementary Materials for cross tabulation tables). For the income analysis, annual incomes greater than $50,000 were excluded from the analysis due to the small number of households in that category. Lower income increased the likelihood of asthma [*χ*^2^(2,*N* = 237) = 7.75, *p* = 0.021], and problems with floors/foundations [*χ*^2^(2,*N* = 237) = 7.78, *p* = 0.02]. Lower income showed a trending association with increased likelihood to have sewage [*χ*^2^(2,*N* = 237) = 4.17, *p* = 0.12] and window [*χ*^2^(2,*N* = 237) = 3.8, *p* = 0.15] problems.

Finally, we examined the association between structural problems and asthma. The analysis revealed that only poor windows and doors [*χ*^2^(1,*N* = 253) = 3.8, *p* = 0.05] was associated with higher prevalence of asthma; roofing (*p* = 0.76), floors (*p* = 0.78) and sewage problems (*p* = 0.73) failed to show an association.

## Discussion

The results of the current study support our hypotheses. Home structural problems, in this case drafty windows and doors, were associated with increased prevalence of respiratory illness (i.e., asthma). The results also showed that living in a manufactured home with repair needs and having low income was associated with increased prevalence of respiratory illness.

The prevalence of asthma and allergies in the sample is drastically higher than the national average. The prevalence rate for asthma in households 100% below the poverty level in the US is 10.4% (18) and 7.7% (adults and children) overall (8). In our sample, 31.6% of the households had at least one person with asthma. Allergies (seasonal allergy, eczema, or food allergy) have a prevalence rate of 31.8% in U.S. adults (20) while in the current sample 56.9% of households had at least one person with allergies. Previous studies have shown that asthma rates are higher in low-income communities (17), therefore the current results are congruent with previous studies. Environmental factors have been found to cause asthma (21). Poverty increases exposure to substandard housing with increased mold (22, 23), air pollution (22, 24), and other triggers that affect asthma (e.g., pests, smoking, etc.) (21). While the current results show an association between income and asthma, we do not find a significant association between income and housing conditions in our sample. This suggests that the environment itself, including the high prevalence of older mobile homes, may interact with the effect of income on housing quality. Further research is necessary to fully examine the effect.

Unlike what has been reported in more urban areas, there is a high rate of home ownership in the predominantly Black rural communities of the Alabama Black Belt. Alabama, which is a predominately rural state, has a higher rate of homeownership (69.7%) than the national average (64.8%) (25). However, half of the homes surveyed were mobile homes. Mobile homes do provide an opportunity for independence; however, in the current study we found that they had an adverse impact on health when they are in disrepair. For example, while income was *not* associated with allergies, living in a mobile home was linked to a higher prevalence of allergies. A recent study reported a similar finding—living in a mobile home increased COVID-19 infection rates (26). We failed to obtain the age of housing units. Given the previous report (11), it may be expected that the manufactured homes in our sample are older homes especially given the repair needs reported. Future research focused on characterizing how aging manufactured homes affect health. It may also be important to characterize how they may be affected by climate change.

Climate change is expected to exacerbate respiratory disease (27). The increase in frequency of storms and heavy rainfall along with the rise in sea levels and increased duration and frequency of floods will result in an increase in dampness and mold problems which leads to increased respiratory problems (21, 22). Additionally, previous studies have reported increased emergency department visits for respiratory conditions after hurricanes (24, 28). By March of 2023 Alabama had the highest number of severe storm reports including tornados in the US (29). The problems associated with climate change may be expected to be catastrophic in the Alabama Black Belt given the current problems with housing structures (e.g., leaky roofs and drafty windows and doors) and soil with poor drainage capacity (7). While repairing homes and improving housing in the region is critical, it is made more difficult by the lack of tradesmen and construction/contractors in the region. A new report by the Associated General Contractors of America reported that more than a quarter of the country's tradesmen is aged over 55 and that there is a severe shortage that is expected to grow in the coming years (30). This shortage has a greater impact in rural communities, especially impoverished rural areas making repairing homes even more difficult. This combination of factors makes it important to monitor respiratory health and introduce interventions to ensure it does not worsen.

There are some limitations to the current study that require attention. The sample is relatively small and has some bias. Because the data were collected as part of a HUD project, the majority of the households surveyed have safety concerns regarding their home. Therefore, there is likely an oversampling of homes with significant repair needs. The self-reported data is also a limitation that may result in misreporting. We failed to collect racial/ethnic data making it difficult to determine whether the sample is representative of the population from which it was sampled. Finally, the data does not allow for an establishment of causation. Future longitudinal studies that also collect data related to other factors linked to asthma (e.g., smoking in the home and exposures to other environmental toxins) are necessary.

## Conclusions

The current study fills an important gap in our understanding of the housing and respiratory health concerns in the Alabama Black Belt region. Asthma and allergy rates are extremely high in the sample studied and may be linked to poor housing conditions. Because of the nonporous soil and the prevalence of storms future research is needed to understand whether excess moisture is contributing to the poor housing conditions and the high rates of respiratory illness particularly for those living in aging mobile homes. There are also potential policy implications including a need to better monitor asthma in the region and providing support to low-income homeowners to ensure homes are safe.

## Data Availability

The raw data supporting the conclusions of this article will be made available by the authors, without undue reservation.

## References

[1] Corley EG Till G O'Brien S Katsinas SG Bray NJ. COVID-19 and Alabama’s Black Belt.

[2] Estrada LV Levasseur JL Maxim A Benavidez GA Pollack Porter KM. Structural racism, place, and COVID-19: a narrative review describing how we prepare for an endemic COVID-19 future. Health Equity. (2022) 6(1):356–66. 10.1089/heq.2021.0190 35651360 PMC9148659

[3] McDonough D. “Housing is Healthcare”: Better Health Through Housing First.

[4] Bryant-Stephens TC Strane D Robinson EK Bhambhani S Kenyon CC. Housing and asthma disparities. J Allergy Clin Immunol. (2021) 148(5):1121–9. 10.1016/j.jaci.2021.09.023 34599980 PMC9809049

[5] Grant T Croce E Matsui EC. Asthma and the social determinants of health. Ann Allergy Asthma Immunol. (2022) 128(1):5–11. 10.1016/j.anai.2021.10.002 34673220 PMC8671352

[6] Hughes HK Matsui EC Tschudy MM Pollack CE Keet CA. Pediatric asthma health disparities: race, hardship, housing, and asthma in a national survey. Acad Pediatr. (2017) 17(2):127–34. 10.1016/j.acap.2016.11.011 27876585 PMC5337434

[7] Holt EW Theall KP Rabito FA. Individual, housing, and neighborhood correlates of asthma among young urban children. J Urban Health. (2013) 90:116–29. 10.1007/s11524-012-9709-3 22689297 PMC3579308

[8] CDC. Most Recent National Data. (2021). Available online at: https://www.cdc.gov/asthma/most_recent_data_states.htm (accessed July 27, 2023).

[9] Shorter C Crane J Pierse N Barnes P Kang J Wickens K Indoor visible mold and mold odor are associated with new-onset childhood wheeze in a dose-dependent manner. Indoor air. (2018) 28(1):6–15. 10.1111/ina.12413 28779500

[10] Mendell MJ Mirer AG Cheung K Tong M Douwes J. Respiratory and allergic health effects of dampness, mold, and dampness-related agents: a review of the epidemiologic evidence. Environ Health Perspect. (2011) 119(6):748–56. 10.1289/ehp.1002410 21269928 PMC3114807

[11] Burbank AJ Hernandez ML Jefferson A Perry TT Phipatanakul W Poole J Environmental justice and allergic disease: a work group report of the AAAAI environmental exposure and respiratory health committee and the diversity, equity and inclusion committee. J Allergy Clin Immunol. (2023) 151(3):656–70. 10.1016/j.jaci.2022.11.025 36584926 PMC9992350

[12] CDC. Most Recent National Asthma Data. (2021). Available online at: https://www.cdc.gov/asthma/most_recent_national_asthma_data.htm (accessed August 3, 2023).

[13] Price T. In their Own Words: Young Black Farmers Share What it Takes to Succeed. The Tennessean. 2022. Available online at: https://www.tennessean.com/in-depth/news/american-south/2022/11/16/in-their-own-words-young-black-farmers-share-what-it-takes-to-succeed/69578434007/ (accessed July 25, 2023).

[14] Carrera JS Flowers CC. Sanitation inequity and the cumulative effects of racism in colorblind public health policies. Am J Econ Sociol. (2018) 77(3-4):941–66. 10.1111/ajes.12242

[15] United States Commission on Civil Rights. Home Ownership for Lower Income Families: A Report on the Racial and Ethnic Impact of the Section 235 Program. Washington, DC: US Government Printing Office (1971).

[16] Ensler P. Separate and Unequal: Black Homeowners of Camden Endure Decades in Shoddy Housing. Alabama Appleseed Center for Law & Justice. Available online at: https://alabamaappleseed.org/news/separate-and-unequal-black-homeowners-of-camden-endure-decades-in-shoddy-housing/ (accessed July 25, 2023).

[17] Carrico A. The State of Manufactured Housing in the Alabama Black Belt. 2019. Available online at: https://prosperitynow.org/blog/state-manufactured-housing-alabama-black-belt (accessed July 25, 2023).

[18] Bownes T Zabawa R. The Impact of Heirs’ Property at the Community Level: the Case Study of the Prairie Farms Resettlement Community in Macon County, AL. The Impact of Heirs’ Property at the Community Level: the Case Study of the Prairie Farms Resettlement Community in Macon County, AL. 2019(SRS-244):29-43.

[19] Katsinas SG Till G Corley EG O'Brien S Courchesne E Bray N. Poverty, Housing, & GDP in Alabama’s Black Belt.

[20] Ng AE Boersma P. Diagnosed Allergic Conditions in Adults: United States, 2021. US Department of Health and Human Services, Centers for Disease Control and Prevention, National Center for Health Statistics; 2023 January 1.

[21] Cockcroft DW. Environmental causes of asthma. Semin Respir Crit Care Med. (2018) 39(1):12–8. 10.1055/s-0037-1606219 29427981

[22] Camacho-Rivera M Kawachi I Bennett GG Subramanian SV. Associations of neighborhood concentrated poverty, neighborhood racial/ethnic composition, and indoor allergen exposures: a cross-sectional analysis of Los Angeles households, 2006–2008. J Urban Health. (2014) 91:661–76. 10.1007/s11524-014-9872-9 24771244 PMC4134442

[23] Rauh VA Landrigan PJ Claudio L. Housing and health: intersection of poverty and environmental exposures. Ann N Y Acad Sci. (2008) 1136(1):276–88. 10.1196/annals.1425.032 18579887

[24] Rentschler J Leonova N. Global air pollution exposure and poverty. Nat Commun. (2023) 14(1):4432. 10.1038/s41467-023-39797-4 37481598 PMC10363163

[25] US Census. Available online at: https://www.census.gov/quickfacts/fact/table/US,AL/SEX255222 (accessed July 20, 2023).

[26] Lowell W Dickerson S Gassman-Pines A Gifford E Rangel M. Racial disparities in COVID-19 case positivity and social context: the role of housing, neighborhood, and health insurance. Hous Policy Debate. (2022) 34(4):443–68. 10.1080/10511482.2022.2104336 PMC11407753 39296307

[27] Covert HH Abdoel Wahid F Wenzel SE Lichtveld MY. Climate change impacts on respiratory health: exposure, vulnerability, and risk. Physiol Rev. (2023) 103(4):2507–22. 10.1152/physrev.00043.2022 37326296

[28] Heslin KC Barrett ML Hensche M Pickens G Ringel JS Karaca Z Effects of hurricanes on emergency department utilization: an analysis across 7 US storms. Disaster Med Public Health Prep. (2021) 15(6):762–9. 10.1017/dmp.2020.281 33023692 PMC8024393

[29] Sebree T. Alabama is #1 for Severe Weather Reports. Available online at: https://www.wsfa.com/2023/03/30/alabama-is-1-severe-weather-reports-2023/ (accessed July 25, 2023).

[30] Associated Builders and Contractors, Inc. Construction Workforce Shortage Tops Half a Million in 2023, Says ABC. Available online at: https://www.abc.org/News-Media/News-Releases/entryid/19777/construction-workforce-shortage-tops-half-a-million-in-2023-says-abc (accessed July 25, 2023).

