# Supplementary material for: The effect of the housing crisis in the Alabama Black Belt on respiratory health
Source: Front Allergy. 2024 Aug 21;5:1413171. doi: 10.3389/falgy.2024.1413171 (PMC11371704; doi:10.3389/falgy.2024.1413171)
Supplement: Supplementary file 1 [file Table1.docx]

**Supplementary Data**

| **Frequency Percent Row Pct Col Pct** | **Table of mobile homes by allergies** | | | |
| --- | --- | --- | --- | --- |
|  | **mobile** | **allergies** | | |
|  |  | **0** | **1** | **Total** |
|  | **0** | 64 | 58 | 122 |
|  |  | 25.50 | 23.11 | 48.61 |
|  |  | 52.46 | 47.54 |  |
|  |  | 58.72 | 40.85 |  |
|  | **1** | 45 | 84 | 129 |
|  |  | 17.93 | 33.47 | 51.39 |
|  |  | 34.88 | 65.12 |  |
|  |  | 41.28 | 59.15 |  |
|  | **Total** | 109 | 142 | 251 |
|  |  | 43.43 | 56.57 | 100.00 |
|  | **Frequency Missing = 2** | | | |

| **Frequency Percent Row Pct Col Pct** | **Table of mobile homes by asthma** | | | |
| --- | --- | --- | --- | --- |
|  | **mobile** | **asthma** | | |
|  |  | **0** | **1** | **Total** |
|  | **0** | 91 | 31 | 122 |
|  |  | 36.25 | 12.35 | 48.61 |
|  |  | 74.59 | 25.41 |  |
|  |  | 52.60 | 39.74 |  |
|  | **1** | 82 | 47 | 129 |
|  |  | 32.67 | 18.73 | 51.39 |
|  |  | 63.57 | 36.43 |  |
|  |  | 47.40 | 60.26 |  |
|  | **Total** | 173 | 78 | 251 |
|  |  | 68.92 | 31.08 | 100.00 |
|  | **Frequency Missing = 2** | | | |

| **Frequency Percent Row Pct Col Pct** | **Table of mobile homes by floors** | | | |
| --- | --- | --- | --- | --- |
|  | **mobile** | **floors** | | |
|  |  | **0** | **1** | **Total** |
|  | **0** | 85 | 37 | 122 |
|  |  | 33.86 | 14.74 | 48.61 |
|  |  | 69.67 | 30.33 |  |
|  |  | 54.84 | 38.54 |  |
|  | **1** | 70 | 59 | 129 |
|  |  | 27.89 | 23.51 | 51.39 |
|  |  | 54.26 | 45.74 |  |
|  |  | 45.16 | 61.46 |  |
|  | **Total** | 155 | 96 | 251 |
|  |  | 61.75 | 38.25 | 100.00 |
|  | **Frequency Missing = 2** | | | |

| **Frequency Percent Row Pct Col Pct** | **Table of income by asthma** | | | |
| --- | --- | --- | --- | --- |
|  | **income** | **asthma** | | |
|  |  | **0** | **1** | **Total** |
|  | **1** | 85 | 51 | 136 |
|  |  | 35.86 | 21.52 | 57.38 |
|  |  | 62.50 | 37.50 |  |
|  |  | 52.15 | 68.92 |  |
|  | **2** | 60 | 21 | 81 |
|  |  | 25.32 | 8.86 | 34.18 |
|  |  | 74.07 | 25.93 |  |
|  |  | 36.81 | 28.38 |  |
|  | **3** | 18 | 2 | 20 |
|  |  | 7.59 | 0.84 | 8.44 |
|  |  | 90.00 | 10.00 |  |
|  |  | 11.04 | 2.70 |  |
|  | **Total** | 163 | 74 | 237 |
|  |  | 68.78 | 31.22 | 100.00 |
|  | **Frequency Missing = 16** | | | |

| **Frequency Percent Row Pct Col Pct** | **Table of income by floors** | | | |
| --- | --- | --- | --- | --- |
|  | **income** | **floors** | | |
|  |  | **0** | **1** | **Total** |
|  | **1** | 74 | 62 | 136 |
|  |  | 31.22 | 26.16 | 57.38 |
|  |  | 54.41 | 45.59 |  |
|  |  | 50.68 | 68.13 |  |
|  | **2** | 56 | 25 | 81 |
|  |  | 23.63 | 10.55 | 34.18 |
|  |  | 69.14 | 30.86 |  |
|  |  | 38.36 | 27.47 |  |
|  | **3** | 16 | 4 | 20 |
|  |  | 6.75 | 1.69 | 8.44 |
|  |  | 80.00 | 20.00 |  |
|  |  | 10.96 | 4.40 |  |
|  | **Total** | 146 | 91 | 237 |
|  |  | 61.60 | 38.40 | 100.00 |
|  | **Frequency Missing = 16** | | | |

| **Frequency Percent Row Pct Col Pct** | **Table of income by sewage** | | | |
| --- | --- | --- | --- | --- |
|  | **income** | **sewage** | | |
|  |  | **0** | **1** | **Total** |
|  | **1** | 117 | 19 | 136 |
|  |  | 49.37 | 8.02 | 57.38 |
|  |  | 86.03 | 13.97 |  |
|  |  | 55.45 | 73.08 |  |
|  | **2** | 74 | 7 | 81 |
|  |  | 31.22 | 2.95 | 34.18 |
|  |  | 91.36 | 8.64 |  |
|  |  | 35.07 | 26.92 |  |
|  | **3** | 20 | 0 | 20 |
|  |  | 8.44 | 0.00 | 8.44 |
|  |  | 100.00 | 0.00 |  |
|  |  | 9.48 | 0.00 |  |
|  | **Total** | 211 | 26 | 237 |
|  |  | 89.03 | 10.97 | 100.00 |
|  | **Frequency Missing = 16** | | | |
